# Concomitant diastolic dysfunction further interferes with cognitive performance in moderate to severe systolic heart failure

**DOI:** 10.1371/journal.pone.0184981

**Published:** 2017-10-04

**Authors:** Mi-Seung Shin, Minjeong An, Sunhwa Kim, Jae Lan Shim, Jin-Kyu Park, JinShil Kim

**Affiliations:** 1 Department of Internal Medicine, Gachon University Gil Medical Center, Incheon, Korea; 2 College of Nursing, Chonnam National University, Gwang-ju, Korea; 3 Department of Nursing, Doowon Technical University, Seoul, Korea; 4 Department of Internal Medicine Hanyang University Medical Center, Seoul, Korea; 5 College of Nursing, Gachon University, Incheon, Korea; Scuola Superiore Sant'Anna, ITALY

## Abstract

**Background:**

Studies of the relevance of cardiac functional markers to cognitive performance in heart failure (HF) have primarily focused on systolic markers. In this study, we examine whether concomitant diastolic dysfunction further interferes with cognitive performance in memory, attention, and executive function in patients with HF.

**Methods and results:**

In this cross-sectional correlational study, 82 patients completed face-to-face interviews for neuropsychological testing for cognitive evaluation. Echocardiographic data were obtained from a review of medical records. Mild to moderate (ejection fraction [EF] ≥ 30%) and severe (EF < 30%) systolic dysfunction were present in 55 (67.1%) and 27 (32.9%) patients, respectively, while 21 (26.3%) had diastolic dysfunction (E/e′ > 15). Those patients who had severe systolic dysfunction had significantly lower attention scores (Digit Span Test [DST] backward, t = 2.62, p = 0.011), while those with concomitant severe diastolic dysfunction had significantly lower verbal fluency (t = 2.84, p = 0.006) and executive function (Korean-Trail Making Test Part B) (t = -2.14, p = 0.036) scores than those without severe diastolic dysfunction. After controlling for age and education, systolic patients with HF with concomitant severe diastolic dysfunction had worse cognitive performance in verbal fluency than those without severe diastolic dysfunction (F = 4.33, p = 0.041, partial eta = 0.057). Concomitant moderate to severe systolic and severe diastolic dysfunction further reduced verbal fluency (F = 8.42, p = 0.005, partial eta = 0.106).

**Conclusions:**

Cognitive performance, particularly executive function, was worse in patients with HF with systolic dysfunction when diastolic dysfunction was concomitantly present. Routine monitoring of and surveillance for diastolic dysfunction and cognitive screening are warranted in the management of patients with HF.

## Introduction

Poor cognitive function is a common comorbid condition in heart failure (HF) that often involves multiple domains, including memory, attention, psychomotor speed, and executive function [1, 2]. The progressive deterioration of HF increases patient susceptibility to mild to moderate vascular dementia levels, which affects a wide range of individuals but approximately one in four with HF [1–3]. Poor cognitive function in HF may preclude adherence to therapeutic recommendations that require intricate cognitive processes for decision-making [4–8]. Previous studies reported adverse outcomes associated with cognitive impairment in HF with functional decline, impaired quality of life, and increased mortality [9–11].

Prolonged hemodynamic and circulatory alterations in HF can induce structural and functional brain damage and are largely believed responsible for cognitive impairment [4, 12, 13]. Among cardiac functional indices in HF, poor cognitive performance occurs more often in patients with systolic dysfunction, with reduced ejection fraction (EF) or cardiac output [2, 4, 14, 15]. For example, cognitive impairment was greater in patients with HF with reduced ejection fraction (HFrEF) than in controls without HF [2, 16]. Cognitive performance–particularly memory function–was the most commonly affected area in elderly patients with HF with significant systolic dysfunction (EF < 30%) [14]. Concurrent systolic and diastolic dysfunction in HF was more likely to increase susceptibility to cognitive impairment, particularly that affecting attention and executive function [4, 17]. Studies that reported the association between echocardiography data and cognitive function have primarily been performed in patients with cardiovascular disease and in Framingham offspring; among the examined indices, EF and/or cardiac output were evaluated in healthy controls or patients with HF with preserved EF (HFpEF) [4]. Few studies have investigated the extent of left ventricular (LV) function as it relates to cognitive function in HF [14–15] or whether concomitant systolic or diastolic dysfunction is more likely to affect performance in several cognitive domains in patients with HF. In particular, the influence of diastolic dysfunction on cognitive function has been addressed less often.

To address gaps in knowledge, this study investigated the relationship between cognitive performance and various indices of systolic and diastolic cardiac function estimated by echocardiography in a sample of patients with HF and whether systolic or concomitant systolic and diastolic dysfunction was associated with cognitive function in HF. Specific aims were to examine: (1) the associations of echocardiographic measures with cognitive function in the domains of memory, attention, and executive function; (2) differences in cognitive performance according to systolic dysfunction severity as determined by EF with a cut-off point of <30% for severe systolic abnormality [18] and diastolic dysfunction as determined by an E/e´ ratio > 15 classified as severe abnormality [19]; and (3) the extent to which the concomitant presence of severe diastolic dysfunction affects cognitive decline. The comparison was made with patients with HF who had systolic dysfunction but no severe diastolic abnormalities.

## Materials and methods

### Study design and procedure

This study used a cross-sectional correlational design to examine the associations between echocardiographic markers and cognitive function as well as the relevance of abnormalities to specific domains of cognitive function. As part of a larger comparative study of cognitive function in patients with HF and other chronic conditions, a subset of patients with HF enrolled from two university-affiliated outpatient clinics were included in this study. After the study obtained approval from the institutional review boards (IRBs) of the university-affiliated hospitals, each patient signed a written informed consent statement before completing a face-to-face interview for cognitive evaluation and demographic and clinical information. The names and numbers of the IRBs were Hanyang University (HYI-15-035-4) and Gachon University Gill Medical Center (GAIRB2015-340). After training and testing for face-to-face interviews for data collection, a graduate nursing student and a clinical coordinator administered neuropsychological tests for the cognitive assessment. Two trained nurses with expertise in cardiovascular care then abstracted the echocardiographic data and other clinical information, including comorbidities, medication, and past and current medical history through a review of medical records.

### Subjects

Patients with HF were eligible if they: (1) were age ≥ 21 years; (2) had a diagnosis of HF with abnormal LV systolic function (EF < 52% for men and < 54% for women) [18]; (3) underwent two-dimensional (2D) and Doppler echocardiography in the 2 years before enrollment; and (4) understood the study protocol, could communicate verbally, and agreed to participate in the study. Patients were excluded if they: (1) had HF with valvular heart disease as the etiology; (2) had documented or current disorders that could induce cognitive impairment, including psychiatric disorders, stroke, dementia, or Alzheimer’s disease; (3) were illiterate; or (4) had conditions that precluded seeing or hearing the study materials.

### Measurements

#### Echocardiographic measures

Results from standard 2D and Doppler echocardiography according to current practice guidelines [20, 21] were obtained from electronic medical records. Measures of interest included both LV systolic and diastolic parameters, respectively EF, cardiac output (CO) (L/min), and fractional shortening (FS); peak velocity of early mitral inflow (E velocity), peak velocity of late mitral inflow (A velocity), early mitral annular velocity (e´), E/e´ ratio, and deceleration time of early mitral inflow (DT) [18, 19, 22]. The American Society of Echocardiography guideline [18] was used to estimate EF (= [end-diastolic volume–end-systolic volume]/end-diastolic volume) and CO (= the stroke volume multiplied by the heart rate) [23]. The ejection fraction was obtained using the modified biplane Simpson’s method from the apical four- and two-chamber views. The left ventricular outflow tract (LVOT) diameter was measured in the parasternal long-axis view in systole. The LVOT velocity time integral (VTI) provides information about blood velocity across systole in centimeters. The area of the LVOT multiplied by the LVOT VTI gives the stroke volume.

Systolic dysfunction was determined by cut-off points of EF, with 41–51% for men and 41–53% for women, 30–40%, and < 30% classified as mildly abnormal, moderately abnormal, and severely abnormal, respectively [18]. Diastolic dysfunction was determined by a cut-off point of septal E/e´ ratio > 15 considered severely abnormal [19].

#### Cognitive function

The Seoul Neuropsychological Screening Battery II is the most commonly used measure of cognitive function in Korea with documented psychometric properties [24]. This multidimensional test battery evaluates cognitive performance in the domains of memory, attention, and executive function. The professional manual guidelines were followed for administering and scoring each domain of cognitive function [24].

The Seoul Verbal Learning Test is a measure of memory consisting of a list of 12 words that is orally presented to patients three times consecutively and then once 20 minutes later. The patients are then asked to recall as many of the words as possible regardless of the order of oral presentation immediately after each of the three trials (immediate recall) and 20 minutes later (delayed recall). Scores range from 0–36 and 0–12, respectively.

Digit Span Tests forward and backward measure attention using the oral presentation of a series of numbers with an increase of one number each time; immediate forward or backward repetition then follows until the numbers are recited in an incorrect order. Forward and backward scores are 0–9 and 0–8 numbers, respectively [25].

The Controlled Oral Word Association (COWA) is a measure of executive function using a verbal fluency test in which patients are asked to generate as many words as possible that begin with a given letter in 60 seconds. This process is consecutively repeated for three letters; the score is the total number of generated words for the three trials. The Korean Trail Making Test for the elderly (KTMT-e) Parts A and B is a measure of executive function. Patients are asked to connect numbers in order, or alternating numbers and days of the week, as fast as possible within 5 minutes in the TMT-e A and B, respectively [26]. The score is the time to completion for each part; a faster completion indicates better frontal lobe/executive function [27].

### Statistical analysis

Descriptive statistics were computed to describe the sample, levels of cognitive function, and echocardiography measures. Normality was tested using the Kolmogorov-Smirnov and Shapiro-Wilk tests. Three variables (i.e., COWA, KTMT-e A and B) significantly deviated from the normal distribution; thus, natural log transformation was performed and the transformed data were used in the data analysis. Pearson’s correlation coefficients were computed to examine the associations of echocardiography measures with each domain of cognitive function in HF. Student’s t-test or a one-way analysis of variance was performed to examine the differences for a specific cognitive function in a comparison of those with versus those without systolic or diastolic abnormalities. To evaluate whether cognitive performance was significantly poorer with concomitant systolic and diastolic dysfunction compared with systolic function only, analysis of covariance (ANCOVA) was also performed with adjustment for age and education. Data analyses were performed using SPSS version 23.0 [28], and the level of significance was set at .05.

## Results

A total of 82 patients with HF with echocardiographic measures participated and completed face-to-face interviews for cognitive evaluation using neuropsychological tests. The mean age of subjects was 65.13 (± 9.80) years. More than half of the participants were men (59.8%). More than half had less than a high school education (52.4%). The median time after the HF diagnosis was 38.60 months, with the most common cause of HF being ischemic heart disease (56.1%), followed by dilated cardiomyopathy (31.7%) and hypertension (8.5%). Based on New York Heart Association classification, 32.9% were in class I (asymptomatic), 37.8% in class II (mild functional limitation), and 29.2% in classes III and IV (moderate to severe functional limitation) (Table 1).

**Table 1:** Demographic and clinical characteristics of heart failure patients with preserved or reduced left ventricular function.

Variables		*N* = 82	
		*n* (%) or *Mean* (SD)	Range
Age, yrs		65.13 (9.80)	37–84
Gender	Men	49.0 (59.8%)	
Marital status	Married	68.0 (82.9%)	
Education, yrs	Edu M(SD)	9.85 (4.08)	0–16
	< 12	43.0 (52.4%)	
	= 12	26.0 (31.7%)	
	> 12	13.0 (15.9%)	
Heart failure duration, months		38.60 (52.63)	0–264
NYHA classes	I	27.0 (32.9%)	
	II	31.0 (37.8%)	
	III	22.0 (26.8%)	
	IV	2.0 (2.4%)	
Etiology	DCM	26.0 (31.7%)	
	ICM	46.0 (56.1%)	
	HTN	7.0 (8.5%)	
	Others	3.0 (3.7%)	
Charlson comorbidity Index		2.29 (1.58)	1–6
Chronic atrial fibrillation (Yes)		11 (13.4%)	
Medication, yes	ACE inhibitor	40.0 (48.8%)	
	ARB	6.0 (7.3%)	
	Beta-blockers	50.0 (61.0%)	
	Loop diuretics	40.0 (48.8%)	
	Statins	45.0 (54.9%)	

NYHA, New York Heart Association; ACE, angiotensin converting enzyme; ARB, angiotensin receptor blocker; SD, standard deviation; DCM, dilated cardiomyopathy; ICM, ischemic cardiomyopathy; HTN, hypertension.

### Associations between echocardiographic measures and cognitive function

Using the LVEF cut-off points of >40% (41–51% for men, 41–53% for women), 30–40%, and <30%, 28 (34.1%), 27 (32.9%), and 27 (32.9%) patients had mild, moderate, or severe systolic dysfunction, respectively. Using a cut-off point of E/e′ > 15, 21 patients (26.3%) had severe diastolic dysfunction. Cognitive function scores were 12.32 (± 5.20) and 3.44 (± 2.25) for immediate and delayed recall memory, 6.49 (± 1.77) and 3.11 (± 1.21) for attention (DST forward and backward), and 14.96 (± 8.28) for verbal fluency (executive function). Most patients who attempted the KTMT-e Parts A and B completed these executive function tests within 300 seconds; 80 (97.8%) completed Part A and 78 (95.1%) completed Part B. The mean times to complete KTMT-e parts and A and B were 33.78 (± 21.98) and 63.35 (± 47.85) seconds, respectively (Table 2).

**Table 2:** Neuropsychological cognitive functioning and echocardiographic measures of the study sample.

Measure			n^a^ (%)	Raw score (M ± SD)	Range
Echo-cardiography scores	Systolic function	LVEF (%)		35.43 ± 8.71	15.00–53.00
		Mild ≥ 41	28 (34.1%)		
		Moderate = 30–40	27 (32.9%)		
		Severe < 30	27 (32.9%)		
		CO (L/min)		3.26 ± 1.20	1.17–6.80
		%FS		18.01 ± 6.12	3.00–35.16
	Diastolic function	Evel (m/s)		0.73 ± 0.70	0.20–6.14
		Avel (m/s)		1.03 ± 1.60	0.40–12.90
		DT (ms)		182.26 ± 43.80	63.00–305.27
		e´ (cm/s)		5.05 ± 2.04	0.03–10.03
		E/e´		12.99 ± 6.06	0.10–38.04
		≤ 15	59 (73.8%)				
		> 15	21 (26.3%)				
Cognitive function	Memory (SVLT)	Immediate Recall		12.32 ± 5.20	2.0–23.0
		Delayed Recall		3.44 ± 2.25	0.0–9.0
	Attention (DST)	Forward		6.49 ± 1.77	3.0–9.0
		Backward		3.11 ± 1.21	0.0–8.0
	Executive function	COWA		14.96 ± 8.28	1.0–42.0
		KTMT-A	80^b^	33.78 ± 21.98	10.0–130.0
		KTMT-B	78^b^	62.35 ± 47.85	5.0–300.0

Note. N = 82.

LVEF, left ventricular ejection fraction; CO, cardiac output; %FS, fractional shortening; Evel, peak velocity of early mitral inflow; Avel, peak velocity of late mitral inflow; DT, deceleration time of early mitral inflow; e´, early mitral annular velocity; E/e, ratio of E per e´; SVLT, Seoul Verbal Learning Test; DST, Digit Span Test; COWA, Controlled Oral Word Association; KTMT, Korean Trail Making Test.

^a ^Numbers vary due to missing data; percentages were estimated based on the available data.

^b ^Persons who successfully completed the test in 300 seconds.

Bivariate correlations between each systolic and diastolic index and the cognitive domains showed no significant correlations between systolic measures and cognitive function. However, mild to moderate associations of most diastolic function indices with cognitive domains existed, with significant correlation coefficients ranging from -0.24 to 0.46. In particular, with an increase in E/e´ ratio, which was used to determine diastolic dysfunction, verbal fluency (r = -0.24, p = 0.036) and executive function (KTMT-e A) (r = 0.27, p = 0.018) decreased (Table 3).

**Table 3:** Associations between echocardiography measures and cognitive functioning.

		Memory				Attention				Executive function					
		SVLT Immediate recall		SVLT Delayed recall		DST forward		DST backward		Verbal fluency COWA^a^		KTMTe-A^a^		KTMTe-B^a^	
		r	p	r	p	r	p	r	p	r	p	r	p	r	p
Systolic Function	LVEF	0.16	0.145	0.16	0.150	0.08	0.460	0.16	0.154	0.09	0.423	-0.05	0.630	0.09	0.445
	CO	0.03	0.817	0.04	0.767	0.01	0.924	0.01	0.965	0.10	0.425	-0.09	0.501	0.12	0.340
	FS	-0.09	0.416	-0.04	0.732	0.05	0.668	0.16	0.147	0.08	0.464	-0.03	0.815	0.07	0.547
Diastolic Function	Evel	-0.02	0.869	-0.10	0.437	-0.09	0.461	-0.18	0.128	-0.06	0.607	0.11	0.359	0.09	0.468
	Avel	0.16	0.215	0.01	0.999	-0.16	0.231	0.01	0.918	0.00	0.992	0.42	0.001	0.41	0.001
	DT	0.46	<0.001	0.40	0.001	0.22	0.076	0.08	0.535	-0.04	0.786	-0.05	0.681	-0.03	0.807
	e´	0.16	0.154	0.11	0.349	0.18	0.124	0.25	0.027	0.24	0.042	-0.26	0.026	-0.06	0.630
	E/e´	0.02	0.878	-0.14	0.232	-0.14	0.223	-0.20	0.073	-0.24	0.036	0.27	0.018	0.19	0.092

Note. N = 82.

SVLT, Seoul Verbal Learning Test; DST, Digit Span Test; COWA, Controlled Oral Word Association; KTMT, Korean Trail Making Test; LVEF, left ventricular ejection fraction; CO = cardiac output; %FS, fractional shortening; Evel, peak velocity of early mitral inflow; Avel, peak velocity of late mitral inflow; DT, deceleration time of early mitral inflow; e´, early mitral annular velocity; E/ e´, ratio of E per e´.

^a ^natural log–transformed data were used.

### Age-and education-adjusted cognitive differences by echocardiographic abnormality

Patients with HF and severe systolic abnormalities (LVEF < 30%) had significantly poorer attention than those with mild to moderate systolic abnormalities (LVEF ≥ 30%) (DST backward, t = 2.62, p = 0.011); however, no significant intergroup differences were found in attention forward, immediate and delayed memory, or executive function. Severe diastolic dysfunction using a cut-off point of an E/e´ ratio >15 was found in 25.3% of patients, with cognitive decline being significantly greater in verbal fluency (t = 2.84, p = 0.006) and executive function (TMT- B, t = -2.14, p = 0.036) than in patients without severe diastolic dysfunction.

To evaluate the impact of concomitant systolic and diastolic dysfunction on cognitive function, ANCOVA was performed with adjustment for age and education. Patients with HF with both risks (mild to severe systolic dysfunction; severe diastolic dysfunction) had significantly poorer cognitive function in verbal fluency than those without severe diastolic dysfunction (F = 4.33, p = 0.041, partial eta = 0.057). The concomitant presence of both risks for cognitive performance was further examined in that patients with moderate to severe systolic (LVEF ≤ 40%) and diastolic (E/e´ > 15) dysfunction (n = 17; 21.2%) had significantly worse verbal fluency than their counterparts with a single risk of moderate to severe (n = 36) or mild (n = 23) systolic dysfunction or mild systolic and severe diastolic dysfunction (n = 4) (F = 8.42, p = 0.005, partial eta = 0.106) (Table 4).

**Table 4:** Age- and education-adjusted neuropsychological cognitive function test scores by significant echocardiography measure in heart failure patients.

Echocardiography measures		No (%) of Patients	Memory (SVLT)				Attention (DST)				Executive function					
			Immediate recall		Delayed recall		Forward		Backward		COWA ^c^		KTMTe-A ^c^		KTMTe-B ^c^	
			t or F	p	t or F	p	t or F	p	t or F	p	t or F	p	t or F	p	t or F	p
LVEF ^a^	≥30^d^	55 (67.9%)	1.26	0.213	1.66	0.101	1.47	0.146	2.62	0.011	0.38	0.706	-0.25	0.800	0.10	0.924
	< 30	26 (32.1%)^e^														
E/e´ ^a^	≤ 15^d^	59 (74.7%)	-0.95	0.346	0.69	0.490	1.38	0.172	1.79	0.077	2.84	0.006	-1.56	0.122	-2.14	0.036
	> 15	20 (25.3%)														
*Both dysfunction ^b^	No^d^	59 (73.8%)	2.24	0.138	0.04	0.835	0.60	0.443	0.89	0.349	4.33	0.041	0.49	0.486	1.87	0.176
	Yes	21 (26.3%)														
**Both dysfunction ^b^	No^d^	63 (78.8%)	1.23	0.272	0.13	0.721	0.02	0.878	0.25	0.616	8.42	0.005	1.20	0.276	0.55	0.459
	Yes	17 (21.2%)														

^a^by t-test

^b^by analysis of covariance, age, and education were controlled as covariates

^c^natural log–transformed data were used

^d^reference group

^e^missing 1 from memory and attention.

*number of patients who had concomitant systolic (mild to severe) and diastolic (E/e´ > 15) dysfunction.

**number of patients who had concomitant moderate to severe systolic (LVEF ≤ 40%) and diastolic (E/e´ > 15) dysfunction.

SVLT, Seoul Verbal Learning Test; DST, Digit Span Test; COWA, Controlled Oral Word Association; KTMT, Korean Trail Making Test; LVEF, left ventricular ejection fraction; E/e´, ratio of E per e´.

## Discussion

This study is one of the few to examine cognitive performance associated with poor cardiac function markers; measures included LV function indices, i.e., EF and E/e´ ratio. The influence of diastolic dysfunction on cognitive performance was also assessed given the reduced systolic function in patients with HF. Patients with HF underwent comprehensive neuropsychological testing to assess the cognitive domains of memory, attention, and executive function. Approximately one-third (26.3%) had combined systolic and diastolic dysfunction, which increased the risk of cognitive decline, particularly for executive function.

Cognitive decline associated with systolic dysfunction was previously reported and primarily focused on reduced EF or cardiac output associated with impaired cognitive performances in multiple domains [2, 4, 6]. Patients with HF with moderate to severe systolic dysfunction (EF ≤ 40) demonstrated significantly poorer cognitive function in multiple domains including attention, memory language, psychomotor speed, and executive function than healthy controls [2, 16] or patients with chronic diseases other than HF [2]. Such impairments–particularly memory loss–appeared to be worse in advanced systolic HF and in patients ≥ 63 years with an EF < 30% [14]. In another study, the pattern of cognitive dysfunction according to EF (markedly reduced, ≤35%; mild, 35–55%; and normal, ≥55%) was nonlinear; individuals with markedly reduced EF showed worse cognitive performance on global, motor speed, and visuoconstructional tests. These impairments were greater in patients with concomitant hypotension, indicated by low mean arterial pressure, than either alone among those with coronary artery disease but without HF who underwent coronary artery graft surgery [15].

The highlight of this study was its demonstration of cognitive decline in patients with HF and concomitant systolic and diastolic dysfunction. Few studies have investigated the extent to which mild to moderate and severe systolic dysfunction, or reduced cardiac output with or without concomitant diastolic dysfunction, was related to poor cognitive function. In this study, diastolic indices had greater influence than systolic indices on cognitive function and decline. Systolic functional indices including LVEF, cardiac output, and FS were not associated with cognitive dysfunction; moreover, mild, moderate, and severe EF reductions showed no significant correlation with any of the cognitive domains. However, most of the diastolic measures showed mild to moderate correlations with some of the cognitive domains. Comparisons of cognitive function according to systolic or diastolic dysfunction revealed differences for attention and/or executive function. Poor cognitive performance was even worse in patients with combined systolic and severe diastolic dysfunction, with a noticeable decrease found in verbal fluency, with adjustment for age and educational level for their influences on diastolic function and/or cognitive performance.

The prognostic implication of severe diastolic dysfunction in patients with HF was previously reported in that prognosis was worse in concomitant systolic and diastolic dysfunction than either alone [29–31]. However, the impact of diastolic dysfunction on cognitive performance in systolic HF has rarely been addressed. Our results provide additional prognostic information about the cognitive impact of diastolic function in patients with HF, particularly those with advanced HF and reduced EF; the concomitant diastolic abnormality further interfered with cognitive performance. Diastolic dysfunction may precede HF with impaired LV systolic function [29, 32]. With worsening HF, diastolic abnormalities also develop. In patients with chronic HF with severe systolic dysfunction, concomitant diastolic dysfunction often occurs due to impaired LV relaxation and elevated filling pressure. One possible explanation for the greater effect of diastolic indices on cognitive performance in this study could be such compromised hemodynamics. Given the circumstance of progressive deterioration of HF with reduced EF, decreased systemic to cerebral perfusion is associated with primarily reduced cardiac output and can induce ischemic damage in the brain [33]. In HF, aging and/or arterial stiffening, particularly aortic stiffening, contributes to increasing left ventricular stiffness, leading to further detrimental effects on systolic and diastolic compliance. Increased ventricular filling pressure also prevents return of the circulating blood to the heart, which viciously alters the systematic to cerebral circulation [34]. Therefore, such a poor hemodynamic alteration, particularly cerebral hypoperfusion in a combination of diastolic dysfunction and systolic HF, may lead to structural and functional changes in the brain by having a detrimental effect on the cerebral small vessels and contribute to the development of cognitive impairment in multiple domains to the level of mild to moderate vascular dementia [1, 34].

Given the presence of systolic HF, the poor cognitive performance, particularly reduced executive function, noted in this study indicates that such impairments are more likely to occur with concomitant severe diastolic dysfunction after the adjustment for age and education. Severe systolic abnormalities (LVEF < 30%) were significantly associated with worse attention (DST backward) than the counterparts of mild to moderate systolic abnormalities, while severe diastolic dysfunction (E/e´ ratio >15) was significantly associated with worse performance in executive function (verbal fluency and trail making test) than the counterparts free of severe diastolic dysfunction. After age and education adjustment, the verbal fluency of those with concomitant diastolic dysfunction remained significantly reduced compared to those with systolic dysfunction (mild to severe) only. Concomitant severe diastolic dysfunction (E/e´ > 15) with moderate to severe systolic dysfunction (LVEF ≤ 40%) further interfered with cognitive performance in verbal fluency compared to their counterparts, a single risk of mild to severe systolic dysfunction only or combined mild systolic and severe diastolic dysfunction.

Our findings were partly consistent with those of a past study, which further supports the predictive value of LV function, particularly diastolic function, in screening for cognitive impairment. In the limited but available data of the functional impact of LV on cognitive function [17], adults aged 59–87 years (EF = 63%) were followed for echocardiography data at baseline (2000–2001) and follow-up (2005–2009) and neuropsychological cognitive evaluation at follow-up. Approximately one-third developed HF (34.5%); of them, 81 (75%) had a reduced EF, the presence of which was associated with worse attention and executive function. In this general population, baseline systolic function was not significantly associated with follow-up cognitive function, while elevated baseline diastolic indices were associated with reduced attention and executive function for a mean 5.9 years of follow-up. At follow-up, most ventricular function abnormalities were associated with lower performance on attention and executive function tests, while their cognitive impacts were insignificant after adjustments for covariates and their baseline values [17].

The results of the two studies were consistent but contradictory, which is probably associated with methodological issues including study design, measures of neuropsychological functioning or target populations, and variations in the cognitive domains of concern. In both studies, cognitive function was decreased in patients with HF, particularly regarding attention and executive function. Cognitive impairment in HF was largely documented with focus on its association with systolic HF, while reduced cognition was reported in a variety of the domains, including memory, attention, language, psychomotor speed, and executive function [2]. The predictive value of LV function, particularly diastolic dysfunction, for worse cognitive function was also consistent in patients with HF as well as the general population, one-third of whom had HF. Additionally, a past study that used a longitudinal design showed the associations of both prior and present LV dysfunction with later cognitive performance, implying the predictive value of the early identification of LV dysfunction associated with cognitive decline [17]. Our study results add evidence of the relationship between systolic and diastolic dysfunction and neuropsychological impairment, particularly in the domains of attention and executive function, which were worse in association with LV abnormalities in HF.

### Limitations

This study has several limitations. First, its results were derived from a small sample of patients with HF, so validation with a larger sample is needed. Given the meager cognitive impacts of systolic indices in this study, particularly reduced EF, the implication of the reduced systolic function should be further examined in a comparison with patients with HFpEF, which has been growing in prevalence and now affects approximately half of all patients with HF [35], while adversity of the reduced systolic function for cognitive performance relative to that in HFpHF remains undetermined. It also remains unknown whether the development of diastolic dysfunction in HF with a reduced EF may further intensify cognitive impairment. The additional impact of diastolic dysfunction on cognitive impairment should be examined in age- and sex-matched groups with varying degrees of diastolic dysfunction. Third, the nature of this cross-sectional study design prevents the understanding of changes in cardiac function and the extent to which cognitive function is affected by the progression of systolic and/or diastolic dysfunction. More empirical evidence is needed to determine whether diastolic dysfunction and cognitive screening can enhance HF management and improve outcomes using a longitudinal study design.

## Conclusions

The findings of this study extend our current knowledge about the influence of severe diastolic dysfunction on cognitive performance in HF with reduced systolic function and the relevance of both systolic and diastolic function on cognitive performance. Given the age-dependent development of diastolic dysfunction [19] with relatively stable systolic indices of EF [18], concomitant diastolic dysfunction was associated with cognitive performance, particularly executive function; this was a significant area of concern, with decline being more likely to occur independent of age and education.

The poor cognitive function associated with diastolic dysfunction in HF and reduced EF observed here implies that periodic cognitive screening and monitoring of diastolic dysfunction should be incorporated in HF management approaches. Such diastolic dysfunction was modifiable through cardiovascular risk control interventions such as weight reduction programs [36, 37]. Studies are warranted to determine whether modifiable factors for diastolic dysfunction in HF could benefit brain fitness and cognitive performance by promoting active engagement in lifelong self-care. The fact that cognitive decline is associated with diastolic dysfunction in systolic HF also requires investigation regarding outcomes.

### Implications

Our study findings have several implications for individual and population-based health care practices for HF management. First, cardiac dysfunction is modifiable; thus, efforts to detect HF deterioration or the development of diastolic dysfunction are needed, particularly for elderly patients with HF. Second, cognitive screening as part of routine care may help identify persons at risk of developing mild to moderate vascular dementia in HF and may prevent or delay cognitive impairment. Future studies are warranted to examine the prognostic implications of diastolic function for cognitive impairment using a longitudinal study design with comparison groups. Third, cognitive function is also modifiable. Improvement can facilitate decision-making for therapeutic recommendations at multiple levels, increasing the likelihood of adherence to the recommendations. Therefore, more data are needed regarding mild cognitive impairment and its adverse effects; and the reversal of cognitive impairments through cognitive training or tailored interventions could improve health outcomes.
